# Differential Regulation of the Three Eukaryotic mRNA Translation Initiation Factor (eIF) 4Gs by the Proteasome

**DOI:** 10.3389/fgene.2019.00254

**Published:** 2019-03-29

**Authors:** Amandine Alard, Catherine Marboeuf, Bertrand Fabre, Christine Jean, Yvan Martineau, Frédéric Lopez, Patrice Vende, Didier Poncet, Robert J. Schneider, Corinne Bousquet, Stéphane Pyronnet

**Affiliations:** ^1^INSERM UMR1037, Centre de Recherche en Cancérologie de Toulouse, Equipe Labellisée Ligue Contre le Cancer and Laboratoire d’Excellence Toulouse Cancer, Université de Toulouse, Toulouse, France; ^2^UMR9198 CEA, Institut de Biologie Intégrative de la Cellule (I2BC), Centre National de la Recherche Scientifique, Université Paris-Sud, Gif-sur-Yvette, France; ^3^School of Medicine, New York University, New York, NY, United States

**Keywords:** mRNA translation, eIF4G, DAP5, PEST, NQO1, NRF2, proteasome, oxidative stress

## Abstract

The 4G family of eukaryotic mRNA translation initiation factors is composed of three members (eIF4GI, eIF4GII, and DAP5). Their specific roles in translation initiation are under intense investigations, but how their respective intracellular amounts are controlled remains poorly understood. Here we show that eIF4GI and eIF4GII exhibit much shorter half-lives than that of DAP5. Both eIF4GI and eIF4GII proteins, but not DAP5, contain computer-predicted PEST motifs in their N-termini conserved across the animal kingdom. They are both sensitive to degradation by the proteasome. Under normal conditions, eIF4GI and eIF4GII are protected from proteasomal destruction through binding to the detoxifying enzyme NQO1 [NAD(P)H:quinone oxidoreductase]. However, when cells are exposed to oxidative stress both eIF4GI and eIF4GII, but not DAP5, are degraded by the proteasome in an N-terminal-dependent manner, and cell viability is more compromised upon silencing of DAP5. These findings indicate that the three eIF4G proteins are differentially regulated by the proteasome and that persistent DAP5 plays a role in cell survival upon oxidative stress.

## Introduction

In eukaryotes, most nuclear encoded mRNAs are modified at their 5′ end with a cap-structure (m7GpppN, where N is any nucleotide). Once mRNA has been exported to the cytoplasm, one function of the cap is to facilitate mRNA translation into protein by the ribosome. Ribosomes are recruited at the mRNA 5′ cap by eIF4F (reviewed in Merrick, 2015), a complex composed of three proteins: the cap-binding protein eIF4E, the RNA-helicase eIF4A and the scaffolding protein eIF4G which interacts with both eIF4E and eIF4A. eIF4G also interacts directly with eIF3, a translation initiation factor bound to the small ribosomal subunit, and with the poly(A) binding protein (PABP). Thus, through multiple interactions, eIF4G plays a central role in cap-dependent translation initiation by bridging the mRNA 5′ cap structure (*via* eIF4E) to the poly(A) tail (*via* PABP), and to the ribosome (*via* eIF3).

Two eIF4G protein homologs have been characterized: eIF4GI and eIF4GII (Gradi et al., 1998). Although both clearly function in translation initiation, they differ in various aspects. Distinct phosphorylation sites targeted by different signaling pathways and with specific biological functions have been mapped in both amino-acid sequences. For instance, both eIF4GI and eIF4GII interact with the MAPK-interacting protein kinases MNK1 (Pyronnet et al., 1999) or MNK2 (Scheper et al., 2001), but only eIF4GI has been described as an MNK1/2 substrate (Orton et al., 2004). A third more distant protein homolog termed DAP5 (also called NAT1, eIF4GIII, or p97) has been identified (Imataka et al., 1997). The DAP5 polypeptide is devoid of N-terminal PABP- and eIF4E-binding sites but possesses domains interacting with eIF4A, eIF3 (Imataka et al., 1997) and MNK1/2 (Pyronnet et al., 1999). Consistently, DAP5 has been implicated in the specific translational regulation of a subset of mRNAs (Lee and McCormick, 2006) and in eIF4E-independent translation when cap-dependent translation is altered such as upon exposure to different stresses (Nevins et al., 2003). The three members of the eIF4G family thus appear to serve as fine regulators of translation initiation under various physiological or stress conditions. However, how the steady state level of each protein is controlled and whether they can be differentially targeted to degradation upon stress remain poorly understood.

## Materials and Methods

### Cell Culture and Compounds

NIH-3T3 cells were grown in standard conditions as described previously (Galés et al., 2003). To obtain cells expressing significantly low levels of DAP5, HEK-293 cells grown in standard conditions were first used to produce viruses upon transfection of the packaging plasmids pPAX2 and pMD2, and a pTRIPZ vector containing a tetracycline-inducible promoter driving the expression of the TurboRFP fluorescent reporter (GE Dharmacon Technology). ShRNAs directed against DAP5 (sh1-DAP5: 5′-TACCTCTAGTAATGGGCTTTA-3′ and sh2-DAP5: 5′-AACCAGCCAAAGCCTTAAATT-3′) or a non-silencing scrambled sequence (shNS: 5′-AATTCTCCGAACGTGTCACGT-3′) were cloned into pTRIPZ using *Eco*RI and *Xho*I restriction sites. NIH-3T3 cells were transduced with cell-free virus-containing supernatants and selected against 4 μg mL^-1^ puromycin during 48 h. RFP-positive and puromycin resistant cells were sorted (MoFlo XDP, Beckman) to establish derivative pools with stable expression of the shRNA constructs. As compared to shNS, the concentration of doxycycline producing an optimal down-regulation of DAP5 in both sh1-DAP5 and sh2-DAP5 cells was 4 μg mL^-1^. Puromycin, doxycycline, cycloheximide, lactacystin, MG-132, dicumarol and H_2_O_2_ were from Sigma and they were dissolved as recommended by the manufacturer.

### MTT Assay

For each condition, triplicates of native or stably transfected NIH-3T3 cells seeded in 96-well plates were let grown for 24 h, treated with or without 4 μg mL^-1^ doxycycline for 48 h and incubated in the absence or presence of H_2_O_2_ for 16 or 24-h. Cell survival was monitored by measuring absorbance at 570 nM with a microplate reader (Mithras-LB-940, Berthold) next to incubation with MTT (Euromedex).

### Plasmids and Transient Transfections

Plasmids (depicted in Figure 2A) used for transient transfections of NIH-3T3 cells were described earlier (Pyronnet et al., 1999) and are as follows: pcDNA3-HA-eIF4GI, pcDNA3-HA-eIF4GII and pcDNA3-HA-DAP5 (encoding full length proteins); pcDNA3-HA-4GI-N and pcDNA3-HA-4GII-N (encoding N-terminal fragments of eIF4GI and eIF4GII). pcDNA3-HA-4GII-C containing the C-terminal two-thirds of eIF4GII was constructed by insertion of a *Eco*RI-*Xho*I PCR fragment amplified from the pcDNA3-HA-eIF4GII using the forward catgacGAATTCcgactttacaccagcctttgct and reverse catgacCTCGAGttttagttatcctcagactcctc primers. The NQO1 expression plasmid was as described previously (Alard et al., 2009). Transient transfections of NIH-3T3 cells was carried out with GeneJet^TM^ (SignaGen Laboratories) according to the manufacturer’s instructions. Proteins were expressed for 36 h and cells were either processed immediately for immunoblotting or processed following treatment with various compounds (where indicated).

### Co-immunoprecipitation and Immunoblot Analyses

Preparation of cell extracts, co-immunoprecipitation and immunoblotting were carried out as previously described (Pyronnet et al., 1999). The antibodies used were as follows: anti-eIF4GI and anti-eIF4GII (gifts of Prof. Nahum Sonenberg); anti-DAP5 (CliniSciences #610742); anti-HA-7 (Sigma); anti-β-tubulin (GeneTex #6288022); anti-4E-BP1, anti-NRF2 and anti-p53 (Cell Signaling Technologies #9452, #12721, and #1C12, respectively); anti-Core 20S (Enzo Life Sciences #PW8155); and anti-NQO1 (Santa Cruz #C19).

### Protein Sequences Analyses

To test for the presence of potential PEST motifs, amino-acid sequences of eIF4GI, eIF4GII and DAP5 from different animal species (described in Table 1) were up-loaded into the ePESTfind software at EMBOSS explorer^1^. After running out the ePESTfind software, only the potential PEST motifs displaying a score >5.0 were taken into account in this study. The alignment showing conservations between eIF4GI and eIF4GII sequences was performed using the LALIGN program^2^.

**Table 1:** Species and accession numbers of the eIF4G family members analyzed.

Species (branch)	Protein name	Accession number
*Home sapiens* (mammals)	eIF4GI	AAI40897.1
	eIF4GII	AAI36644.1
	DAP5	AAI11416.1
*Gallus gallus* (birds)	eIF4GI	XP_015147015.1
	eIF4GII	XP_015152751.1
	DAP5	NP_001093330.1
*Alligator sinensis* (reptiles)	eIF4GI	XP_014383304.1
	eIF4GII	XP_025059396
	DAP5	XP_025052452.1
*Xenopus tropicalis* (amphibians)	eIF4GI	XP_012818831.1
	eIF4GII	NP_001135544.1
	DAP5	NP_001004992.1
*Danio rerio* (fishes)	eIF4GI	NP_001073669.1
	eIF4GII	XP_021325628.1
	DAP5	NP_001014311.1
*Drosophila melanogaster* (insects)	eIF4GI	AAF59403.3
	eIF4GII	AAF56194.2
	DAP5	AAF58443.2


## Results

### eIF4GI and eIF4GII Exhibit Shorter Half-Lives Than DAP5

**FIGURE 1:**
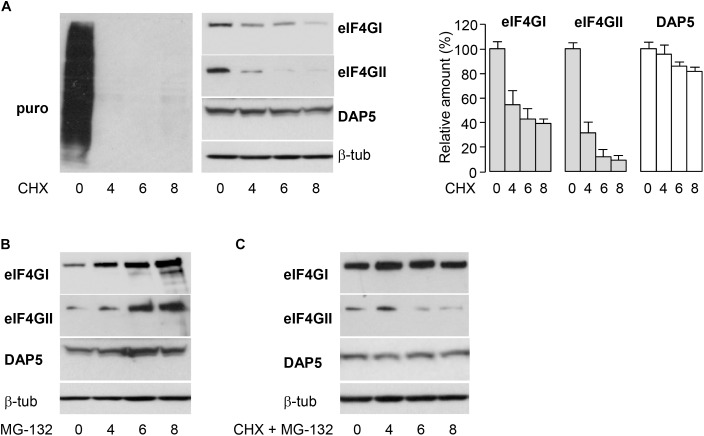
Differential half-lives of eIF4GI, eIF4GII and DAP5. **(A)** NIH-3T3 cells were treated at different times with 50 μg mL^-1^ cycloheximide (CHX) and incubated with 10 μg mL^-1^ puromycin (puro) 10 min before lysis. Proteins were visualized by western-blotting with the indicated antibodies (left and middle) and signals were quantified by densitometric analysis (right). Data are the means ± SD of three separate experiments. **(B)** NIH-3T3 cells were treated at different times with 20 μM MG-132 and proteins visualized by western-blotting as indicated. **(C)** NIH-3T3 cells were co-treated at different times with 50 μg mL^-1^ CHX and 20 mM MG-132 and proteins visualized by western-blotting as indicated.

To explore how the amounts of the three different eIF4G family members are controlled, their respective half-lives were first monitored in non-transformed NIH-3T3 fibroblasts. Cells were treated with cycloheximide (CHX) to arrest protein synthesis and time-dependent decreases in the cellular contents of eIF4GI, eIF4GII, and DAP5 were visualized by western-blotting. Protein synthesis was efficiently blocked by CHX as attested by a metabolic labeling of cells with puromycin and its subsequent detection into nascent polypeptides by western-blotting (Figure 1A, left). Both eIF4GI and eIF4GII exhibited short half-lives (∼5 and ∼3 h, respectively) as compared to DAP5 which remained unaltered after 8 h of treatment with CHX (Figure 1A, middle and right). One probable explanation for the relative short half-lives of eIF4GI and eIF4GII in cells is that they are rapidly degraded by the proteasome. Consistently, eIF4GI has been shown earlier to be a proteasomal substrate with a relative short half-life (Baugh and Pilipenko, 2004). We therefore tested this possibility by incubating cells with MG-132, a specific proteasome inhibitor. Both eIF4GI and eIF4GII markedly accumulated in cells following treatment with MG-132 while the amount of DAP5 increased only slightly over the same period (Figure 1B). Furthermore, the fast decrease in eIF4GI or eIF4GII amount observed upon inhibition of protein synthesis by CHX was less pronounced when cells were co-incubated with MG-132 (Figure 1C). These data indicate that eIF4GI and eIF4GII exhibit faster turnovers than DAP5 in growing fibroblasts likely due to more rapid degradation by the proteasome (and probably higher rates of synthesis).

### Involvement of PEST-Containing eIF4GI or eIF4GII N-Terminus in Proteasomal Degradation

**FIGURE 2:**
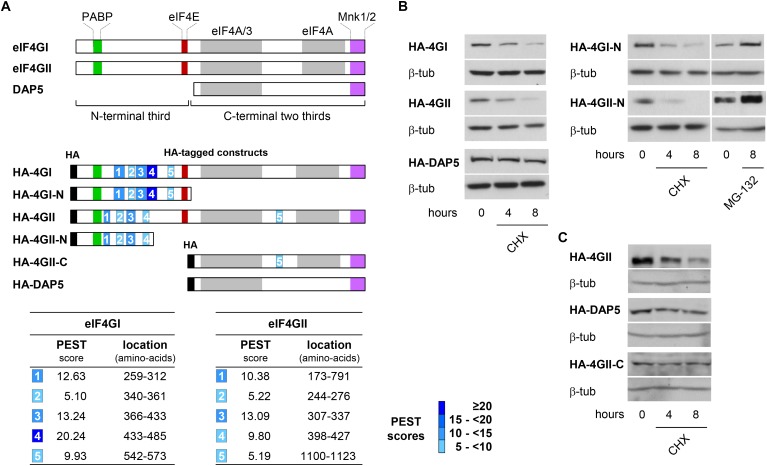
Identification of PEST regulatory motifs in eIF4GI and eIF4GII N-termini. **(A)** Schematic representation of eIF4G protein family members highlighting the binding domains with their main partners (colored boxes) and N-terminal third and C-terminal two thirds of eIF4GI and eIF4GII proteins (brackets) (top). Schematic representation of HA-tagged (black boxes) full-length and deletion fragments used for transient transfections. The computer-predicted (ePEST-find, EMBOSS) PEST motifs detected in eIF4GI and eIF4GII are numbered 1–5 (middle). Scores and locations of the five PESTs found in eIF4GI and eIF4GII polypeptides (bottom). **(B)** Following transfection with HA-tagged full-length (left) or N-terminal (right) cDNAs, NIH-3T3 cells were untreated or treated at different times with 50 μg mL^-1^ CHX or MG-132 and proteins visualized by western-blotting as indicated. **(C)** Following transfection with HA-eIF4GII (top), HA-DAP5 (middle), or HA-eIF4GII-C (bottom) cDNAs, NIH-3T3 cells were untreated or treated at different times with 50 μg mL^-1^ CHX and proteins visualized by western-blotting as indicated.

We then searched how eIF4GI, eIF4GII, and DAP5 are differentially targeted to the proteasome for degradation. eIF4GI and eIF4GII are considered as two functional homologs because they both contain domains interacting with key translation initiation factors (including PABP, eIF4E, eIF3, and eIF4A) and with the translation regulators MNK1 and MNK2 kinases. The more distant homolog DAP5 shows similarities only with the C-terminal two thirds of eIF4GI or eIF4GII as it lacks the N-terminal third and as a consequence cannot interact with PABP or eIF4E (Figure 2A, top). We therefore suspected that N-terminal features shared only by eIF4GI and eIF4GII could be responsible for their more rapid turnovers. *In silico* predictions (ePESTfind, EMBOSS) revealed the existence of five putative PEST-motifs (sequences enriched in proline, glutamate, serine and threonine) with variable scores in each eIF4GI and eIF4GII polypeptides. Similar computer-predicted PEST motifs were identified earlier in the eIF4GI amino acid sequence (Anand and Gruppuso, 2005). Five and four PEST motifs were detected in the N-terminal thirds of eIF4GI and eIF4GII, respectively, while one with a low score was found in the C-terminal two-thirds of eIF4GII (Figure 2A, middle and bottom). In contrast, no sequences reaching computer-predicted PEST requirements could be detected in the DAP5 polypeptide. PEST-motifs are known to target proteins for degradation by the proteasome either dependently on or independently of ubiquitination (Mathes et al., 2008). They have been found and validated in other key short-lived proteins including c-MYC (Gregory and Hann, 2000), members of the I-kappaB family (Lin et al., 1996; Park et al., 2014) and ornithine decarboxylase (ODC) (Ghoda et al., 1992). To test for their implication in the fast turnovers of eIF4GI and eIF4GII, experiments designed to monitor protein half-lives and similar to those described in Figure 1 were repeated using HA-tagged full-length eIF4GI, eIF4GII, and DAP5 (named HA-4GI, HA-4GII, and HA-DAP5; Figure 2A, middle), or HA-tagged N-terminal segments of eIF4GI and eIF4GII each containing 5 or 4 PEST motifs (named HA-4GI-N and HA-4GII-N; Figure 2A, middle). Following transfection and CHX treatment, HA-4GI and HA-4GII exhibited half-lives as short as those observed for the endogenous proteins, while HA-DAP5 devoid of PEST motifs remained unaltered (Figure 2B, left). In addition, HA-4GI-N and HA-4GII-N were also similarly short-lived upon inhibition of protein synthesis by CHX, but accumulated upon inhibition of proteasomal activity by MG-132 (Figure 2B, right). In contrast, HA-4GII-C containing the C-terminal two-thirds of eIF4GII but lacking its N-terminal third was more resistant to degradation upon CHX treatment, and showed a stability similar to that of HA-DAP5 (Figure 2C). These data suggest that PEST-containing N-terminal thirds of eIF4GI and eIF4GII form signals sufficient for targeting them to proteasomal degradation.

### DAP5 Is Resistant to Degradation Under Oxidative Stress

**FIGURE 3:**
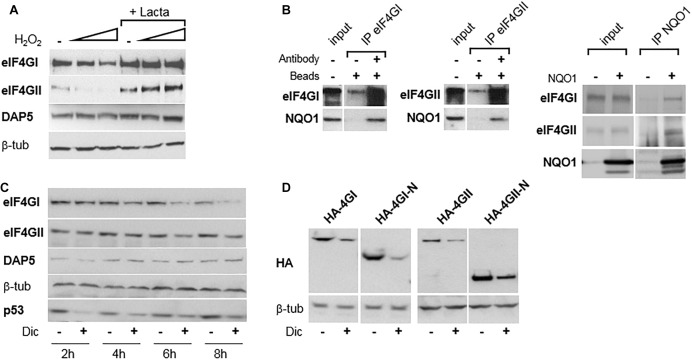
eIF4GI and eIF4GII, but not DAP5, are degraded under oxidative stress. **(A)** NIH-3T3 cells were untreated or treated with increasing concentration of H_2_O_2_ in the presence or absence of lactacystin (described in Alard et al., 2009), and protein extracts were subjected to western-blotting as indicated. **(B)** NIH-3T3 cell extracts were subjected to western-blotting with the indicated antibodies either directly (input) or after immunoprecipitation (IP) with either eIF4GI or eIF4GII antibodies (left). NIH-3T3 extracts of cells either untransfected of transfected with NQO1 cDNA were subjected to western-blotting with the indicated antibodies either directly (input) or after immunoprecipitation (IP) with NQO1 antibodies (right). **(C)** NIH-3T3 cells were untreated or treated with 300 μM dicumarol (Dic) at different times and proteins were visualized by western-blotting as indicated. **(D)** Following transfection with HA-tagged, full-length or N-terminal cDNAs, NIH-3T3 cells were untreated or treated with 300 μM dicumarol for 8 h and proteins visualized by western-blotting as indicated.

eIF4GI can be destructed directly by the 20S proteasome (Baugh and Pilipenko, 2004) and we (Alard et al., 2009) and others (Attar-Schneider et al., 2014) have shown that it is protected from degradation through its binding to NQO1, an observation made initially for the two other short-lived proteins p53 (Asher et al., 2003) and ODC (Asher et al., 2005); and more recently extended to other key proteins including HIF-1α (Oh et al., 2016). NQO1 protects candidate proteins from degradation by the proteasome through its direct interaction with the 20S proteasome (Moscovitz et al., 2012). However, upon oxidative stress which recruits NQO1 and its quinone oxidoreductase activity for detoxifying reactive oxygen species, eIF4GI (and other protected proteins) no longer binds to NQO1 and becomes more rapidly degraded by the proteasome (Alard et al., 2009). To check whether a similar degradation of eIF4GII or DAP5 occurs, the fate of both proteins was monitored under oxidative stress. Increasing concentrations of H_2_O_2_ resulted in a degradation of eIF4GII sensitive to the proteasome inhibitor lactacystin, while the amount of DAP5 remained unchanged (Figure 3A). This suggested that eIF4GII is subjected to a similar mechanism of deregulation than that of eIF4GI under oxidative stress. The possibility that eIF4GII also interacts with NQO1 has been therefore verified. Co-immunoprecipitation experiments confirmed the interaction between eIF4GI and NQO1 (Figure 3B, left), and revealed that eIF4GII similarly interacts with NQO1 when co-immunoprecipitation is performed with either anti-eIF4GII (Figure 3B, middle) or anti-NQO1 (Figure 3C, right) antibodies.

The protective binding of NQO1 to eIF4GI (Alard et al., 2009), p53 (Asher et al., 2003), and ODC (Asher et al., 2005) is disrupted by dicumarol, an NQO1-specific inhibitor which provokes the accumulation of intracellularly produced ROS. Consistently, incubation of cells with dicumarol provoked the degradation of eIF4GI and eIF4GII, while the amount of DAP5 was not affected (Figure 3C). p53 was used here as positive control of protein degradation induced by dicumarol (Figure 3C), although its degradation and recovery followed faster kinetics than those of eIF4GI or eIF4GII. In addition, HA-tagged full-length as well as N-terminal thirds of both eIF4GI and eIF4GII were all degraded next to dicumarol treatment (Figure 3D), indicating that PEST-containing N-terminal domains of the two homologs are sufficient to mediate proteasomal degradation under oxidative stress.

### DAP5 Is Involved in Cell Survival Under Oxidative Stress

**FIGURE 4:**
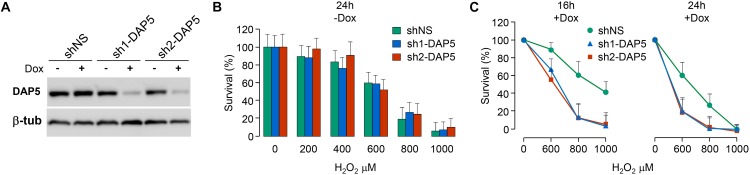
DAP5 is involved in cell survival under oxidative stress. **(A)** NIH-3T3 cells stably transfected with non-specific (NS) or DAP5-specific (sh1-DAP5 or sh2-DAP5) inducible shRNAs were untreated or treated with doxycycline for 48 h and protein extracts were subjected to western-blotting with the indicated antibodies. **(B)** The survival of stably transfected NIH-3T3 cells in the absence of Dox but treated for 24 h with H_2_O_2_ was monitored as a function of H_2_O_2_ concentration. Data are the means ± SD of three separate experiments and normalized (%) to the values obtained with H_2_O_2_-untreated cells. **(C)** The survival of stably transfected NIH-3T3 cells incubated with Dox for 48 h and treated for 16 or 24 h with H_2_O_2_ was monitored as a function of H_2_O_2_ concentration. Data are the means ± SD of five separate experiment and normalized (%) to the values obtained with H_2_O_2_-untreated cells.

Because DAP5, but not eIF4GI or eIF4GII, was unaffected by oxidative stress, it was probable that this translation initiation factor was involved in the cellular response to oxidative stress. This hypothesis was tested by using three pools of NIH-3T3 fibroblasts, two of them engineered to express distinct doxycycline-inducible shRNAs directed against DAP5 (named sh1-DAP5 and sh2-DAP5) and one to express scrambled shRNAs (named shNS). As compared to the shNS pool, the treatment of cells with doxycycline for 48 h efficiently down-regulated DAP5 protein expression in sh1-DAP5 and sh2-DAP5 pools (Figure 4A). Before testing the possible involvement of DAP5, survival of the three pools of NIH-3T3 cells under oxidative stress was first monitored in the absence of doxycycline to ensure that the processes of antibiotic selection and cell sorting (see section “Materials and Methods”) did not generate a pool of cells with an intrinsically (i.e., independent of DAP5 amount) distinct response to oxidative stress. A dose effect of H_2_O_2_ during 24 h confirmed that survival of the three pools of cells was similarly affected by oxidative stress (Figure 4B). Then, this experiment was repeated but in the presence (or not) of doxycycline for 48 h to down-regulate DAP5 expression followed by treatment with H_2_O_2_ during 16 or 24 h. The data clearly showed that DAP5 down-regulation altered cell survival under oxidative stress at both times tested (Figure 4C).

### NRF2 or NQO1 Expressions Is Independent of DAP5 Under Oxidative Stress

**FIGURE 5:**
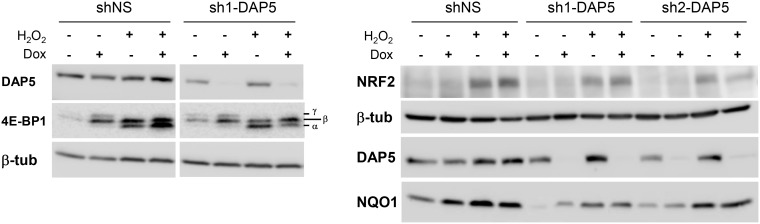
Induction of NRF2 and NQO1 proteins under oxidative stress is independent of DAP5. Protein extracts of stably transfected NIH-3T3 cells grown in the absence or presence of doxycycline (Dox) for 48 h and untreated or treated with 1 mM H_2_O_2_ for 4 h were subjected to western-blotting with the indicated antibodies. The bottom-to-top α–β–γ symbols denote hypo- to hyperphosphorylated 4E-BP1 isoforms.

One important factor induced by and required for the response to oxidative stress is NRF2 (recently reviewed in Bellezza et al., 2018). NRF2 induces the transcriptional activation of genes capable of detoxifying intracellular ROS, including NQO1 which acts through its quinone oxidoreductase activity (Venugopal and Jaiswal, 1996). We therefore hypothesized that NRF2 and/or NQO1 expression could be altered at the translational level upon down-regulation of DAP5. This assumption was supported by the fact that oxidative stress is known to inhibit general cap-dependent translation initiation while DAP5 is believed to play a role in cap-independent translation under stress (Nevins et al., 2003), and that a cap-independent mode of NRF2 mRNA translation has been described upon oxidative stress(Li et al., 2010). The impact of oxidative stress on cap-dependent translation was first looked in shNS and in sh2-DAP5 cells in the absence or presence of doxycycline. In both cell pools, H_2_O_2_ treatment provoked a significant dephosphorylation of 4E-BP1, as shown by accumulation of its α hypophosphorylated isoform (Figure 5, left). As hypophosphorylated 4E-BP1 sequesters the cap-binding translation initiation factor eIF4E, this supported the notion that cap-dependent translation is actually inhibited in our cell models, and that NRF2 (and likely NQO1) expression could be controlled by DAP5 in a cap-independent manner. The experiment was therefore repeated with the three pools of cells untreated or treated with doxycycline. Both NRF2 and NQO1 expressions were actually induced upon treatment with H_2_O_2_, but such inductions were not affected by DAP5 down-regulation in either of the two sh1-DAP5 and sh2-DAP5 stable cell lines (Figure 5, right). These data revealed that although required for cell survival, DAP5 is not involved in NRF2 or NQO1 protein induction under oxidative stress.

## Discussion

**FIGURE 6:**
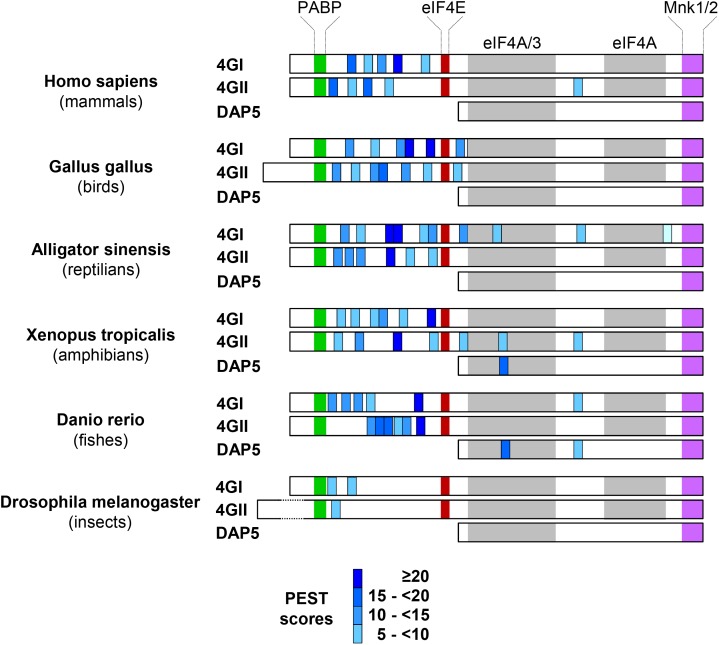
Conservation of N-terminal eIF4Gs’ PEST motifs among different branches of the animal kingdom. The ePESTfind software was run out for eIF4GI, eIF4GII, or DAP5 proteins from representative species of main branches of the animal kingdom. The color code is as in Figure 1 except that PEST motifs are not numbered. Note that for convenience, the colored boxes corresponding to the binding domains of PAPB, eIF4E, eIF4A, eIF3, and MNK1/2 validated in the mammalian protein sequences have been copy/pasted in protein sequences of other species even if they have not been always experimentally confirmed.

These data indicate that the intracellular amounts of the three eIF4G family members are differentially regulated by the proteasome. The DAP5 polypeptide devoid of N-terminal PEST motifs is more stable than eIF4GI and eIF4GII proteins. Curiously, the N-terminal segments of eIF4GI and eIF4GII containing the functional PEST motifs are the less conserved portions among the two proteins (Supplementary Figure S1). The domain structures of eIF4GI and eIF4GII have been extensively studied. As compared to their well characterized C-terminal two-thirds, no folded domains have been identified in their N-terminal thirds although individual shorter stretches of amino-acids may fold upon binding to their respective partners such as PABP and eIF4E (Marintchev and Wagner, 2005), and likely NQO1 (Figure 3B). The N-terminal third of eIF4GI or eIF4GII can therefore be viewed as an intrinsically disordered and flexible segment allowing changes in conformational states rendering eIF4GI or eIF4GII capable of creating numerous contacts with different proteins involved in protein synthesis. Intrinsically disordered portions of proteins have been often considered as probable signals for proteasomal degradation (Suskiewicz et al., 2011). It is thus possible that the apparently non-conserved but PEST-containing N-terminal segment of eIF4GI or eIF4GII has yet evolved to serve a dual function: (i) it provides a necessary flexibility for assembly of translation initiation complexes and (ii) it forms a signal for proteasomal degradation when not protected by binding partners. Consistently, proteasomal degradation of eIF4GI and eIF4GII coincides with disruption of their binding to NQO1 and to eIF4E (next to 4E-BP1 hypophosphorylation; Figure 5 and Alard et al., 2009), and likely with disruption of their binding to PABP as oxidative stress leads to nuclear re-localization of the protein where it is not expected to interact with eIF4GI or eIF4GII (Salaun et al., 2010). Interestingly, the presence of PEST sequences in the N-terminal third of eIF4GI or eIF4GII is a feature conserved in animal species belonging to different branches of the animal kingdom. Indeed while only anecdotic (i.e., non-conserved among species) and low score PEST motifs were detected in eIF4GI or eIF4GII C-terminal two thirds or in DAP5 polypeptides, eIF4GI or eIF4GII N-terminal thirds of all species contain PEST motifs including those with the highest scores (Figure 6).

Our data also indicate that persistent DAP5 is involved in cell survival upon oxidative stress, although neither NRF2 nor NQO1 expression is affected by DAP5 knock-down. Whether DAP5 still plays a role in the expression of a subset of genes through a selective translational mechanism under oxidative stress remains to be elucidated. If this is the case, how DAP5 could function under oxidative stress? Clues may arise from what happens when cells are exposed to other stresses such as hypoxia. We (Azar et al., 2013) and others (Koritzinsky et al., 2006) have actually shown that hypoxia blocks cap-dependent mRNA translation through eIF4E sequestration by the hypophosphorylated forms of 4E-BP1 and blocks global mRNA translation through eIF2α phosphorylation. However, it has been shown recently that DAP5 selectively recruits the ribosome to target mRNAs under hypoxia *via* its direct interaction with eIF2β (Liberman et al., 2015; Bryant et al., 2018), thus circumventing the inhibitory effect of eIF4E sequestration on cap-dependent translation and the inhibitory effect of eIF2α phosphorylation on global translation. Together with eIF2β and eIF2γ, eIF2α belongs to the eIF2 trimeric translation initiation complex whose function in translation (i.e., binding of the charged tRNA to the small ribosomal subunit) is inhibited next to eIF2α phosphorylation (reviewed in Lasfargues et al., 2013). Since H_2_O_2_-induced oxidative stress also provokes eIF2α phosphorylation (MacCallum et al., 2006) and eIF4E sequestration while sparing DAP5 (our data), a similar DAP5-eIF2β-dependent selective translational mechanism may occur. Additionally, DAP5 may stimulate eIF4E-independent translation initiation of a specific subset of mRNAs by recruiting the ribosome through its very recently described direct interaction with eIF3 (de la Parra et al., 2018).

## Author Contributions

AA and SP designed the experiments and wrote the manuscript. AA, BF, CM, FL, and PV performed the experiments. CB, CJ, DP, RS, and YM helped in writing and edited the manuscript. SP conceived and supervised the project. All authors have reviewed and approved the final manuscript.

## Conflict of Interest Statement

The authors declare that the research was conducted in the absence of any commercial or financial relationships that could be construed as a potential conflict of interest.

## Footnotes

1 http://emboss.bioinformatics.nl/cgi-bin/emboss/epestfind

2 https://embnet.vital-it.ch/software/LALIGN_form.html

## Supplementary Material

FIGURE S1 The PEST motifs reside into the less conserved regions of eIF4GI and eIF4GII. The PABP and eIF4E binding domains are highlighted in green and red, respectively. The PEST motifs are highlighted in light blue. The values appearing next to accession numbers refer to the first and last amino acids (aa) of eIF4GI or eIF4GII protein sequences used to create the alignment. “.” stands for similar amino acids; “:” stands for identical amino acids.
