# Supplementary figures and images for: Differential Regulation of the Three Eukaryotic mRNA Translation Initiation Factor (eIF) 4Gs by the Proteasome
Source: Front Genet. 2019 Mar 29;10:254. doi: 10.3389/fgene.2019.00254 (PMC6449437; doi:10.3389/fgene.2019.00254)

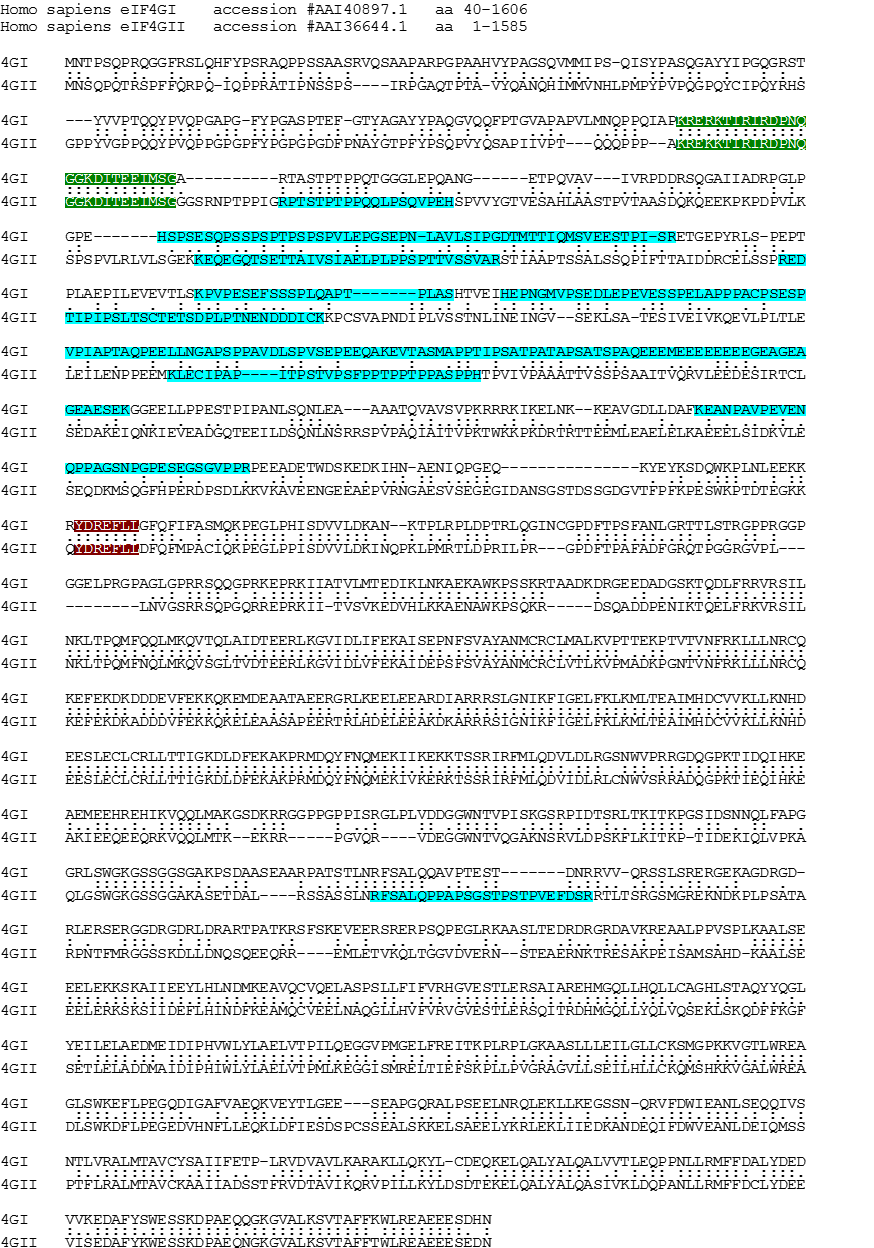

Supplement: FIGURE S1 — The PEST motifs reside into the less conserved regions of eIF4GI and eIF4GII. The PABP and eIF4E binding domains are highlighted in green and red, respectively. The PEST motifs are highlighted in light blue. The values appearing next to accession numbers refer to the first and last amino acids (aa) of eIF4GI or eIF4GII protein sequences used to create the alignment. “.” stands for similar amino acids; “:” stands for identical amino acids. [file Image_1.tif]
